# Effectiveness of the Young-Laplace equation at nanoscale

**DOI:** 10.1038/srep23936

**Published:** 2016-04-01

**Authors:** Hailong Liu, Guoxin Cao

**Affiliations:** 1 HEDPS, Center for Applied Physics and Technology, College of Engineering, Peking University, Beijing 100871, China

## Abstract

Using molecular dynamics (MD) simulations, a new approach based on the behavior of pressurized water out of a nanopore (1.3–2.7 nm) in a flat plate is developed to calculate the relationship between the water surface curvature and the pressure difference across water surface. It is found that the water surface curvature is inversely proportional to the pressure difference across surface at nanoscale, and this relationship will be effective for different pore size, temperature, and even for electrolyte solutions. Based on the present results, we cannot only effectively determine the surface tension of water and the effects of temperature or electrolyte ions on the surface tension, but also show that the Young-Laplace (Y-L) equation is valid at nanoscale. In addition, the contact angle of water with the hydrophilic material can be further calculated by the relationship between the critical instable pressure of water surface (burst pressure) and nanopore size. Combining with the infiltration behavior of water into hydrophobic microchannels, the contact angle of water at nanoscale can be more accurately determined by measuring the critical pressure causing the instability of water surface, based on which the uncertainty of measuring the contact angle of water at nanoscale is highly reduced.

The behavior of liquids at nano-environments has attracted extensive research investigations thanks to its important applications in nanopipets^1^, programmable catalysis^2^, bimolecular detection and separation^3^, water desalination^4^, nanofluidic battery^5^, nanofluidic damper^6^ and so on. Most of the aforementioned important applications are closely related to the pressure-driven infiltration behavior of liquids into nanochannels. For example, the mechanism of nanofluidic damper is that the liquid is pushed into the nonwetting nanopore by the external impact, and thus, the work done by external impact will be converted into the solid-liquid interfacial energy to realize the function of energy-damping^7^,^8^,^9^.

At microscale, the Y-L equation is used to define the equilibrium of liquid surface (e.g., capillary surface)^10^:


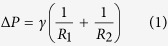


where *γ* is the liquid surface tension (for liquid-air surface), Δ*P* is the pressure difference across the liquid surface, and *R*_*1*_ and *R*_*2*_ are the principal radii of surface curvature. In addition, in a sufficiently narrow tube (circular shape with the radius *a*), the liquid surface will be a portion of spherical surface (with radius *R*) and *R* is related to *a* by the liquid-solid contact angle *θ* (*R* = *a*/cos *θ*). Thus, the Y-L equation can be modified as^11^:


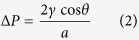


The surface tension represents the potential energy change caused by per unit liquid surface area change, which is typically considered to be a constant, e.g., the *γ* of water is measured as 72 mN/m at room temperature. It is also reported that the surface tension of water decreases with the temperature^12^,^13^, and slightly increases with the electrolyte ion concentration (e.g., 74.0 mN/m for 1.0 M KCl^14^). Currently, the Y-L equation is also widely used to describe the capillary pressure when the channel/pore size is down to nanoscale^15^,^16^,^17^,^18^,^19^.

There is a significant scatter in the experimental results of contact angle at nanoscale since the contact angle is a quantity defined at macroscale, which is difficult to be accurately measured at nanoscale and also very sensitive to the surface quality. Consequently, it is challenging to accurately measure the surface tension at nanoscale. Currently, most investigations about surface tension or contact angle are based on the MD simulations of the behavior of nanobubble/nanodroplet. However, the reported results are highly in conflict: Matsumoto and Tanaka^20^ reported that the surface tension is independent of the surface curvature on the basis of the nanobubble in the Lennard-Jones (L-J) liquid, whereas Nejad H.R. *et al.*^18^ and Park *et al.*^21^ shown that the surface tension of nano-bubble increases with the surface curvature. However, the MD simulations of nanodroplets of L-J liquid shown that the surface tension decreases with the increase of surface curvature.^22^ Homman *et al.*^23^ recently shown that the water surface tension has a non-monotonic curvature dependence from MD simulations. The MD simulations of curved octane-water interface by Sodt *et al.*^24^ demonstrated that the water surface tension is not size dependent. Therefore, it is still not clear whether the Y-L equation is effective at nanoscale or not.

Based on the MD simulations of the infiltration behavior of water into single-walled carbon nanotubes (SWCNT), it was found that the infiltration pressure (Δ*P*) increases with the decrease of the tube radius (*a*) but Δ*P* is not linearly scale with 1/*a* as shown by Equation (2) 25,26,27,28 Walther *et al.* further reported that the infiltration pressure determined in their MD simulations is consistent with that estimated from the Y-L equation.^19^ However, Mo *et al.*^29^ recently reported that the Y-L equation is invalid for nanochannels, which is also based on MD simulations. Therefore, a systematic investigation on the effectiveness of Y-L equation at nanoscale is highly necessary.

In the present work, the effectiveness of Y-L equation at nanoscale is checked using MD simulations, and the influences of surface size, temperature and electrolyte ions on the water surface property at nanoscale are also considered. Figure 1 shows the schematic of the approach used to validate the effectiveness of Y-L equation at nanoscale in the present work: the water is enclosed inside a reservoir and the top of reservoir is covered by a plate with a nanopore. With the increase of the reservoir pressure (*P*_*a*_), the curvature of water surface over nanopore will be changed. The relationship between surface curvature and reservoir pressure is calculated using MD simulations. Figure 2 shows the MD computational model used in the present work: a water reservoir (4.7 × 4.7 × 8.1 nm) includes 6000 water molecules confined between two rigid carbon-atom planes, and the periodic boundary is applied along the lateral directions (*x*, *y*). The reservoir center density (*ρ*) is about 0.997 g/cm^3^. There is a circular hole (with radius of *a* = 1.3–2.7 nm) in the center of the top plane which is fixed, and the bottom plane (also called piston) is moveable along *z*-direction to adjust the internal pressure of reservoir. Initially, the hole in the top plane is covered by a lid, and the system is equilibrated under the selected pressure using NVT ensemble for 200 ps. The Nose-Hoover thermostat^30^ is used to keep the temperature at 298 K and the time integration step is set to 2 fs. After the reservoir is equilibrated, the lid covered on the hole in the top plane is removed and the system is equilibrated for another 1 ns. Both the pressure of the water reservoir (*P*_*a*_) and the number of water molecules coming outside the reservoir as well as their finial equilibrated locations (outside of reservoir) are monitored. For each pressure increment (Δ*P*_*a*_), the piston moves by 0.02 nm till the water molecules burst out of reservoir (i.e., the water molecules on the top of hole lose their stable conformation, see Fig. 2(e,f)).

The MD simulations are carried out using LAMMPS^31^, which is a classical molecular dynamics software from Sandia National Laboratory. The nonbond interactions between water molecules are modeled by the Lennard-Jones (L-J) potential and the Coulombic potential. The SPC/E model^32^ is used to simulate water molecules, which has been shown to provide the best agreement with the experimental value of water surface tension^33^,^34^,^35^. The SHAKE program is used to constrain the internal geometry of water molecules^36^. The long range Columbic potential is calculated using the particle-particle particle-mesh (PPPM) method^37^. The nonbond interaction between carbon-plane and water molecules are modeled by the L-J potential, which is simplified as the C-O nonbond interaction since the C-H nonbond interaction is very small. Two sets of the L-J parameters are used: set A (*ε*_*OC*_ = 0.114333 kcal/mol and *σ*_*OC*_ = 0.32751 nm) representing the hydrophilic surface and set B (*ε*_*OC*_ = 0.07493 kcal/mol and *σ*_*OC*_ = 0.319 nm) representing the hydrophobic surface^38^,^39^. The cut-off distance for the VDW interaction is set to be 1.2 nm which is considered to be accurately describe the VDW interaction of water in the MD simulations^40^,^41^. The simulation results are not sensitive to the reservoir size used in the present study, which has been checked by a larger reservoir (7.0 × 7.0 × 7.3 nm) including 12000 water molecules.

In the present model, the pressure outside reservoir is zero, and thus, the pressure across the water surface (the water molecules face the pore of top plane) is equal to the water pressure in reservoir (Δ*P* = *P*_*a*_). The reservoir pressure can be determined by the water density change from the equation of state of water (derived from bulk water) or is approximated as the piston pressure (as shown in Figure S1). The MD simulation results shown that the curvature radius of water surface will be reversely proportional to the water pressure in reservoir (see Fig. 3(a)). Two principle curvature radii *R*_*1*_ (measured in *xz* plane) and *R*_*2*_ (measured in *yz* plane) of water surface can be measured in MD simulations by the average distribution of water molecules located at the surface (as shown in Figure S2). The Δ*P*-*R* relationship can be fitted as a function similar to Equation (1) (displayed as the dashed line in the Fig. 3a), where *R* is average radius of curvature (1/*R* = (1/*R*_*1*_ + 1/*R*_*2*_)/2).The calculated values of surface tension of water from the different pore sizes and reservoir pressures based on Equation (1) are shown in Fig. 3b. The mean value of surface tension of water *γ* = 62.6 mN/m (displayed as the dashed line in Fig. 3b) and the standard deviation is 0.8 mN/m (as shown in Figure S3), which clearly shows that there is no size dependence of *γ* when the pore size is even down to 1-2 nm. Therefore, it is seen that the Y-L equation is valid at nanoscale from the MD simulations.

In order to further show the present result of *γ* is the intrinsic property of water, we changed the L-J potential parameters of the top plane (with pore) to make it a typical hydrophobic material (using the set B L-J parameters), which gives the contact angle of 110° for the water on graphene (the set A gives the contact angle of 65°)^42^. With the new top plane, the relationship between Δ*P* and *R* is still well fitted by Equation (1), as shown in Figure S4, the dashed line in the figure is the fitting function of Equation (1) with *γ* = 62.6 mN/m. In addition, we also changed the circular pore shape into elliptical shape, as shown in Fig. 3b. By doing so, the principle curvature radii *R*_*1*_ and *R*_*2*_ are different, whereas the average curvature radius *R* is still inversely proportional to Δ*P* (displayed in Figure S4). The calculated *γ* from the elliptical pore as well as from the hydrophobic top plane are very close to the value shown in Fig. 3b (see Figure S5). When the top plane changes from hydrophilic to hydrophobic (the reported contact angle increases from 65° to 110°) and the pore shape changes from circular to elliptical, the variation in the calculated surface tension is less than 5%. Thus, the surface tension calculated in the present work is considered to be the intrinsic property of water, which is not dependent upon the pore size, pore shape as well as the material property of top plane.

The present result of *γ* matches very well with the reported values of the surface tension of bulk water calculated based on MD simulations using the SPC/E water model: *γ* = 61.3 mN/m by Chen *et al.* and *γ* = 62 mN/m by Wynveen *et al.*^33^,^43^. The surface tension calculated by the SPC/E water model is still about 15% lower than its experimental counterpart, which might be caused by the difference between the modeled water and real water. For example, the SPC/E model is nonpolarizable^44^, and it overestimates the diffusion constant of water, which means that the hydrogen bonding is not sufficiently simulated by the model^45^. Actually, besides the SPC/E model, most water models developed in MD simulations, including TIP3P, SPC, TIP4P, TIP5P, and TIP6P models, will give a lower surface tension than its experimental counterpart^33^,^46^. Nevertheless, this difference will not affect effectiveness of our observation: the Y-L equation (Equation (1)) is effective at nanoscale and the surface tension of water is not dependent upon the surface size.

The experimental results show that the water surface tension will decrease with the temperature and increase with the electrolyte concentration^33^,^47^. To investigate the effects of temperature, we changed the aforementioned MD simulation models to higher temperatures. The Δ*P*-*R* relationships of water surface under the different temperatures still follows the function similar to Equation (1), i.e., Δ*P* is inversely proportional to *R*, as shown in Fig. 4a. The calculated values of *γ*under different temperature are shown in Fig. 4b, in which the pore size *a* = 1.7 nm. With the increase of temperature, the surface tension of water decreases. The *γ* under different temperatures obtained in the present work are very close to the corresponding values of bulk water reported by Chen *et al.*^33^ (displayed as the circular symbols in Fig. 4b) and the trend of *γ* varying with temperature in our work is also same as the experimental counterpart (displayed as the square symbols in Fig. 4b)^47^. Therefore, the effect of temperature on the surface tension of water is also independent with the surface size.

The ions of Na^+^ and Cl^−^ are added into water reservoir to simulate the NaCl electrolyte solution. After adding the ions, the surface curvature radius *R* is still inversely proportional to the reservoir pressure, but the *R* of the NaCl solution is larger than that of pure water under a given pressure, as shown in Fig. 4a (the mass concentration of NaCl is 18.5%), which means that the surface tension of the NaCl solution is higher than that of pure water. The calculated *γ* is 74.5 ± 1.0 mN/m (displayed as the diamond symbol in Fig. 4b), which is also slightly lower than its experimental counterpart (78 ± 1 mN/m^48^). Therefore, our approach can also accurately predict the effect of ions on the surface tension of water.

At molecular level, the surface tension is created by the molecular energy difference between the surface molecule and its bulk counterpart. For water, it is commonly considered that the surface tension closely depends upon the distortion of the hydrogen bond between the surface water molecules. The hydrogen bond for water is typically described by the average near O-O distance of water (<0.35 nm) and the H-O-O angle (<30°)^49^. Since the rotation of the water molecule is much easier than the translation in the simulations (i.e. with less potential change), the hydrogen bond strength is considered to be approximately scale with the average near O-O distance of water (the effect of H-O-O angle variation is neglected here). The O-O distance of water molecules will increase with temperature, and thus, the hydrogen bond between water molecules will be weaker. When the water surface curvature changes, the energy increase caused by the distortion of a weaker hydrogen bond will be also lower at a higher temperature, which means that the water surface tension is lower. Adding the electrolyte ions into water, the liquid-liquid interaction energy will increase thanks to the electrostatic interaction between ions, which can be easily seen from its higher frozen and boiling points. Therefore, the energy increase created by the distortion of a stronger interaction will be higher, which means that the water surface tension is higher for the electrolyte solution (NaCl + H_2_O).

When the surface curvature is down to a couple nanometers, it is commonly observed that the hydrogen bond of surface water molecules has a higher distortion due to a larger curvature, which can be shown by the lower coordination number for a lower curvature radius compared with that of bulk water. Based on this analysis, the hydrogen bond of the surface water molecules with a higher surface curvature will be weaker, i.e., the near O-O distance of water molecules will be larger. For example, it is reported that the water molecules infiltrated into nanopore have a higher volume or a larger near O-O distance^50^. In the present MD simulation results, we also found that the coordination number (the number of the nearest neighbors of the molecule) increases with the surface curvature radius (see Fig. 5a, the solid line in the figure is the linear fitting curve, and the slope is about 0.007/Å), and it will converge to the corresponding value of flat surface (~2) when *R* approaches to infinite. However, the near O-O distance of surface water molecules is not sensitive to the surface curvature, as shown in Fig. 5b. The average values of the near O-O distance are determined as ~2.73 Å, which is very close to that of flat surface. It should be noted that there is a thickness of water surface at nanoscale, which is defined from the outside boundary of water molecules (see Figure S2) to the location with the converged near O-O distance (or the coordination number). Cross the surface thickness from outside to inside, the water density rapidly increases, the coordination number of water molecules also increases but the near O-O distance decreases. Finally, the coordination number and the near O-O distance converge to ~2.4 and ~2.7 Å, respectively. Consequently, the near O-O distance (or the coordination number) of water surface is defined as the average values of the whole surface region, and the details are shown in Figure S6 (or Figure S7).

The results show that the trend of the near O-O distance varying with the surface curvature is different than that of the coordination number. The main reason for this divergence is from the pressure compensation. Actually, there is a higher reservoir pressure corresponding to a lower curvature radius (see Fig. 3a). Under a higher pressure, the near O-O distance between water molecules in bulk state will decrease (i.e., a higher pressure with a lower near O-O distance), and then, a higher surface curvature will cause a larger expansion of the near O-O distance of surface due to a small coordination number (i.e., a larger curvature leads to a larger O-O distance). When these two effects are balanced with each other, the O-O distance of surface water molecules will be insensitive to the surface curvature. Therefore, the surface tension of water is not dependent upon the surface curvature and the Y-L equation (Equation (1)) is effective at nanoscale.

We compared our results with the reported results about the size effect of surface tension, most of which are mainly based on the MD simulations of nanobubble or nanodrops built by the L-J liquid^18^,^20^,^21^,^22^,^23^,^24^. In these works showing the conflict results of curvature dependence of surface tension, the surface tension is typically calculated from the integration of the gradient of the normal component of the pressure tensor of liquid molecules and the radius of the nanobubble/nanodrop. However, the effectiveness of using the local pressure tensor as the internal pressure might be problematic when the size of bubble/drop is very small, which has been also discussed by Sampayo *et al.*^51^ In addition, the curved molecular surface of small bubble/drop under no-constraint condition is rarely well defined.^18^,^22^ In our method, the surface tension can be simply determined from the reservoir pressure (or piston pressure) and the curvature of water surface (a small part of a sphere), both of which can be determined with a very low uncertainty (see Figures S2 and S3).

The water surface curvature will continually increase with Δ*P* till the reservoir pressure reaches some critical value Δ*P*_*c*_ (defined as the burst pressure), at which the water surface will lose its stability (see Fig. 2(e,f)). The burst pressure can be determined from the sudden increase of the number of water molecules outside of the stable surface (*N*_*out*_), as shown in Figure S8. Figure 6 shows the relationship between the burst pressure and the nanopore radius, which shows the perfect inversely proportional relationship: Δ*P*=*k*/*a* (the fitting parameter *k* = 104.5 mN/m), displayed as the solid line in the figure. It is surprisingly see that the Δ*P*-*a* relationship follows the Y-L equation (Equation (2)) for the hydrophilic plate with pore, which is conventionally used to describe the infiltration pressure of water into hydrophobic nanochannel. From the surface equilibrium condition at the critical point (see Fig. 1), the fitting parameter *k* = 2*γ*sin *θ*_*c*_, and using the value of *γ* we have determined (*γ* = 62.6 mN/m), it is found that the critical angle *θ*_*c*_ = 56.5°. Actually, the stability of the water surface over the nanopores is kept not only by the surface tension but also closely related to the pin effect of top carbon plane which is described by *γ*_*SG*_* − γ*_*SL*_ = *γ·*cos *θ*_*c*_ (as shown in Fig. 1) and the surface instability will occur when the solid-liquid contact point cannot be pinned, i.e., *γ*_*SG − *_*γ*_*SL*_ > *γ·*cos *θ* when *θ* > *θ*_*c*_. Thus, the critical angle *θ*_*c*_ should be the contact angle between carbon plane and water, and the following relationship can be derived:


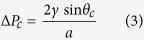


which is actually a modification form of the Y-L equation (Equation (2)).

The contact angle of the water droplet (radius of 4.24 nm) on carbon plate is reported as 65.4° based on MD simulation (with the same L-J parameters as set A used in the present work) by Werder *et al.*^42^ which is about 15% higher than the value determined in the present work. However, they also shown that the contact angle determined varies with their droplet size (i.e., contact angle is not a constant at nanoscale). In addition, there is spacing between water molecules and carbon plate due to the van der Waals repulsion (describe by L-J parameters). Since the contact angle is determined by the tangential line at the cross point of the circular line with the carbon plate in their work, they need to extrapolate the circular line (best fitting the droplet shape) to the carbon plate, which will increase the uncertainty of contact angle. However, in our approach, the contact angle is determined only by the burst pressure and the surface tension determined previously, both of which can be determined with a very low uncertainty. The most important thing is that the contact angle is actually not sensitive to the pore size (*a*) in the present work. Therefore, the contact angle determined from our approach is more stable, which is an intrinsic property of water.

In order to further show the validness of Equation (3), we simulated the burst pressures of water with high temperatures and the NaCl solution (with the mass concentration of 18.46%)as well as the burst pressure of an elliptical pore. The relationships between the burst pressure Δ*P* and pore size *a* of all above cases perfectly follow the inversely proportional relationship: 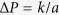, as shown in Fig. 7 (the burst pressure of an elliptical pore is displayed as the circular symbol in Fig. 6), and the lines in the figure are linear fitting function curves. If plugging the values of the surface tension (displayed in Fig. 4b), the contact angles of water with different temperatures as well as the NaCl solution can be also calculated. For example, for the pore size *a* = 1.7 nm, the contact angle *θ*_*c*_ = 70.9° for the present NaCl solution and *θ*_*c*_ = 56–57.5°when the water temperature is in the range of 283–353 K. Therefore, the contact angle will be increased by adding the electrolyte ions and it is not very sensitive to *T* in the present temperature range, which also match the experimental results^52^,^53^. Friedman *et al.*^52^ reported that the contact angle of water on graphite/quartz is actually insensitive to the temperature when *T* < 80 °C, and Zhang *et al.*^53^ shown that the contact angle of NaCl solution increases with the increase of concentration.

When the top plane is changed to the hydrophobic material (using set B L-J parameters corresponding the reported contact angle of 110°), the case is significantly different. There is no critical pressure to cause the instability of water surface. The value of Δ*P* reaches the maximum value at *θ* = 90°, and then the value of *N*_*out*_ rapidly increase. Actually, it is straightforward to see that the maximum surface curvature will be obtained at *θ* = 90° for a given pore size (i.e., the vertical portion of surface tension reaches the maximum), which also means that the surface tension cannot balance the further increase of Δ*P*, and thus, the surface begins to move. However, the solid-liquid contact point is still well pinned since the contact angle *θ*_*c*_ > 90°. Therefore, the present approach only can be used to calculate the contact angle of hydrophilic material (i.e., *θ*_*c*_ < 90°). Actually, the contact angle of hydrophobic material can be estimated by the infiltration pressure of water into hydrophobic microchannel^19^. In the present work, the infiltration pressure (*P*_*in*_) of water into a rigid SWCNT (modeled by set B L-J parameter, which means that the present nanotube is hydrophobic) is also determined using MD simulations, as show in Figure S9. As shown in the inset figure of Figure S9, the water reservoir is connected with a SWCNT, the reservoir pressure *P*_*a*_ increases with the piston displacement, and *P*_*a*_ does not vary with the piston displacement when *P*_*a*_ reaches some critical value (*P*_*in*_), which is defined as the infiltration pressure. Plugging the values of *P*_*in*_, *a* and γ (62.6 mN/m) into the Y-L equation (Equation (2)), the contact angle of SWCNT (with the set B L-J parameter) can be calculated as *θ*_*c*_ = 112°, which is very close to the reported value^42^. Therefore, the contact angle of water with hydrophilic/hydrophobic surface can be effectively determined by measuring the burst pressure of a hydrophilic pore (Fig. 2) or the infiltration pressure of a hydrophobic microchannel (Figure S9).

In summary, a new model including a water reservoir covered with a solid plate with a circular pore is established in the present work, based on which the surface tension of water can be calculated using MD simulations. It is found that the applied reservoir pressure is inversely proportional to the curvature of water surface created over the nanopore, and the fitting parameter of this relationship corresponds to the water surface tension based on the Young–Laplace equation (Equation (1)). The results also show that the relationship holds for the different pore size, pore material, pore shape and water temperature as well as the electrolyte solution (NaCl solution). Thus, our approach can be effectively used to calculate the surface tension of water surface with different size, under different temperature as well as for the electrolyte solutions. In addition, for the hydrophilic solid plate (with pore), there is a well-defined relationship between the burst pressure (causing the instability of water surface) and the contact angle of water with the solid plate, which is similar to the Y-L equation (Equation (2)). Thus, our approach can be further effectively used to calculate the contact angle of water (with different size, different temperature or adding electrolyte ions) on the solid plate (hydrophilic type). Combining with the pressure-driven infiltration behavior of water into hydrophobic microchannels, the contact angle is actually determined by measuring the critical pressure causing the instability of water surface (e.g., the burst pressure or the infiltration pressure). By doing so, the uncertainty in determining the contact angle is highly reduced, and it is also found that the contact angle does not depend on the pore size (indicating an intrinsic property).

In the present work, the investigated nanopore radius range is 1.3–2.7 nm, but our method can be effective for an even larger pore size range. The accuracy of the surface tension determined using the present method actually depends on the accuracies of the measured values of surface curvature (decreases with the pore size) and reservoir pressure (decreases with the increase of pore size). Therefore, the lower-limit of the effective pore radius is decided by the lower-limit of the liquid surface curvature which can be accurately determined (this value is around 0.5 nm).

Finally, the most important thing is that our approach can provide an effective way to check the effectiveness of the Y-L equations (including both equations (1) and (2)) at nanoscale, which is much more stable (with lower uncertainty) than the conventional approach based on the behavior of nanobubble/nanodroplet. Therefore, the present work provided an important guideline to help us to understand the liquid behavior at nanoscale.

## Additional Information

**How to cite this article**: Liu, H. and Cao, G. Effectiveness of the Young-Laplace equation at nanoscale. *Sci. Rep.* **6**, 23936; doi: 10.1038/srep23936 (2016).

## Supplementary Material

Supplementary Information

## Figures and Tables

**Figure 1:**
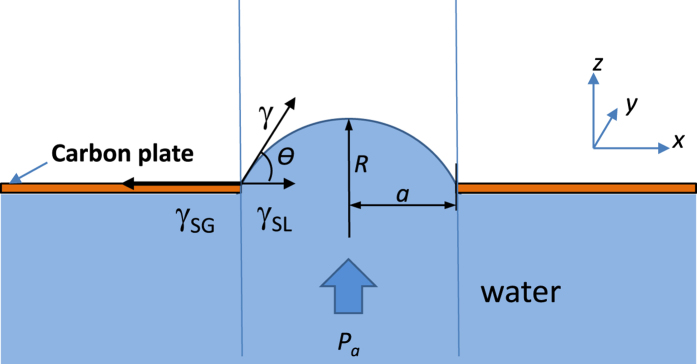
The schematic of the model used to validate the effectiveness of Y-L equation at nanoscale. *a* is the pore radius, *R* is the radius of curvature of water surface, *P*_*a*_ is the reservoir pressure.

**Figure 2:**
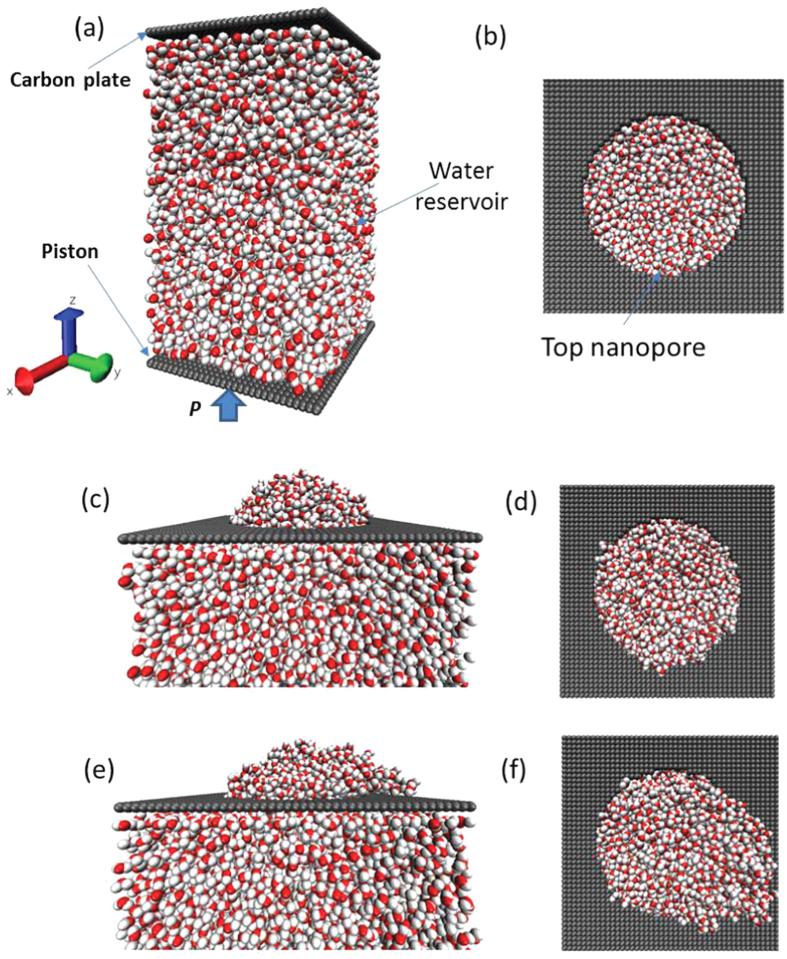
The MD simulation models of the water conformation under the different pressure. (**a,b**) the initial water structure at *P*_*a*_ = 0; (**c,d**) the stable water surface at *P*_*a*_ > 0; (**e,f**) the unstable water surface at *P*_*b*_ (*P*_*b*_ is the burst pressure). (**a,c,e**) are side views and (**b,d,f**) are top views.

**Figure 3:**
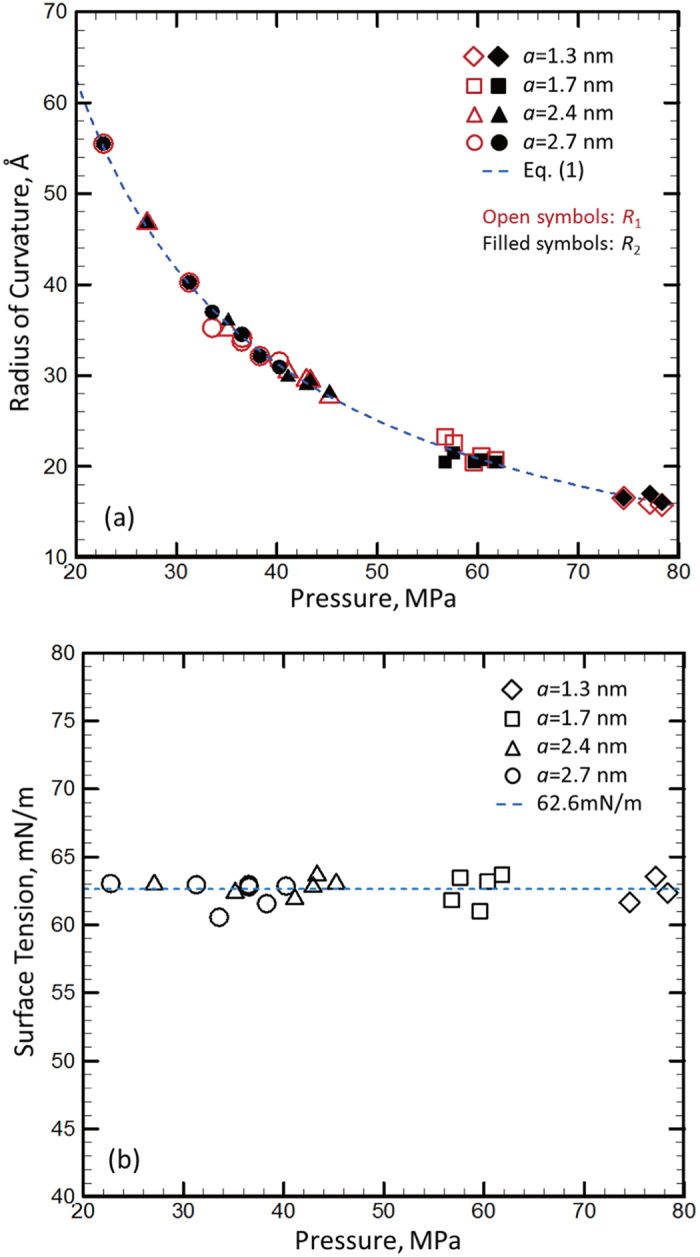
(**a**) The principal radii of curvature of water surface calculated by MD simulations varying with the reservoir pressure. (**b**) The surface tension values calculated from the data displayed in (**a**) using equation (1).

**Figure 4:**
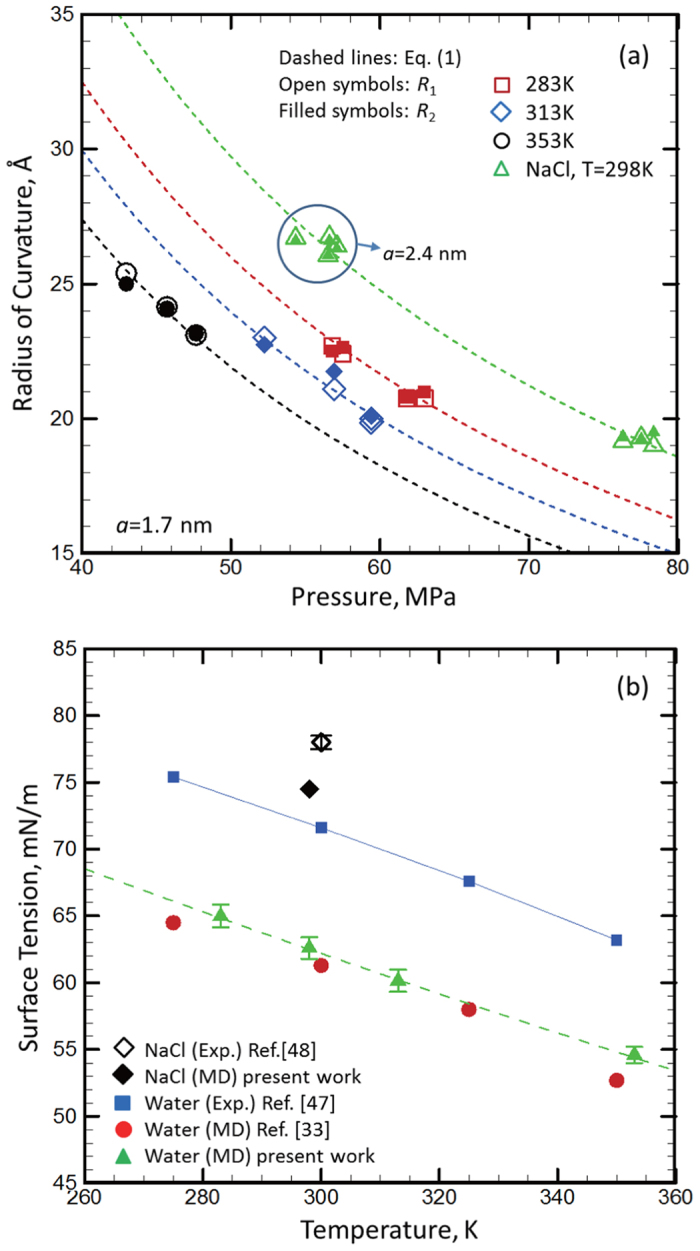
(**a**) The calculated principal radii of curvature of water surface varying with the applied pressure at different temperature as well as with adding electrolyte ions (NaCl). The mass concentration of NaCl is 18.5%. (**b**) The calculated water surface tension based on the data displayed in (**a**) using Equation (1).

**Figure 5:**
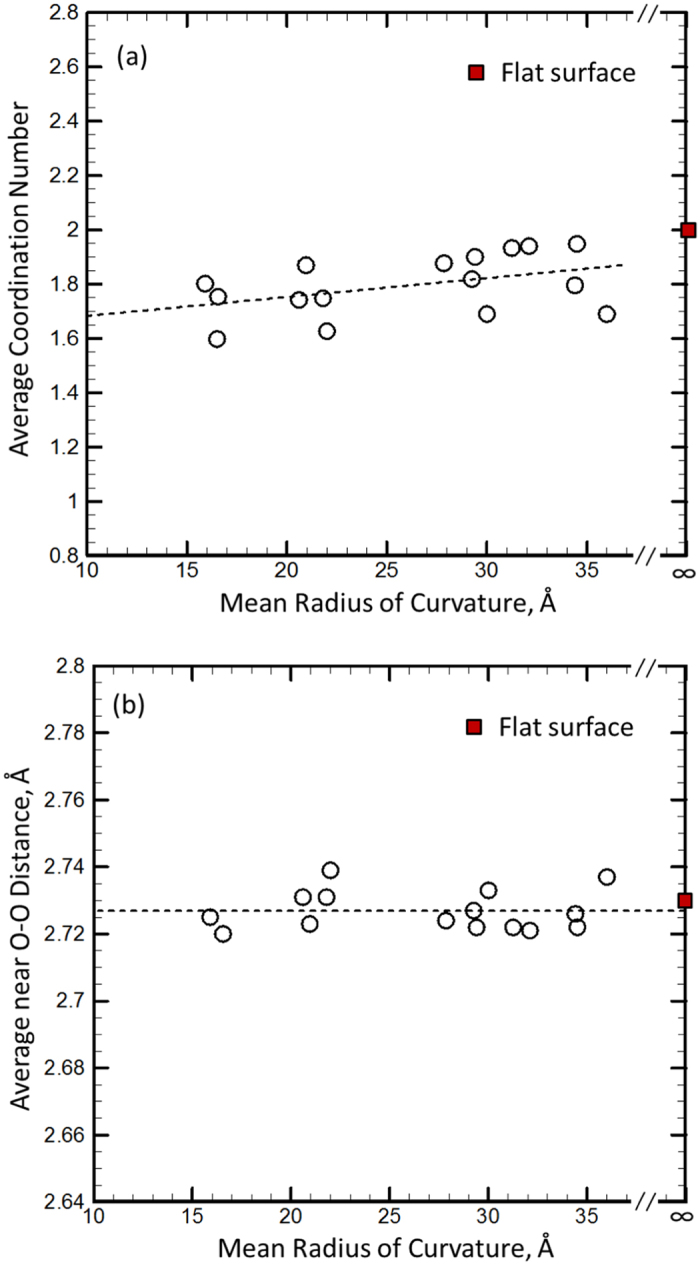
(**a**) The average coordination number of water surface varying with the mean radius of curvature of surface; (**b**) The average near O-O distance of water surface varying with the mean radius of curvature of surface. The dashed lines are the linear fitting functions in the figure. The square symbol represents the results of the infinite curvature radius (i.e., flat surface).

**Figure 6:**
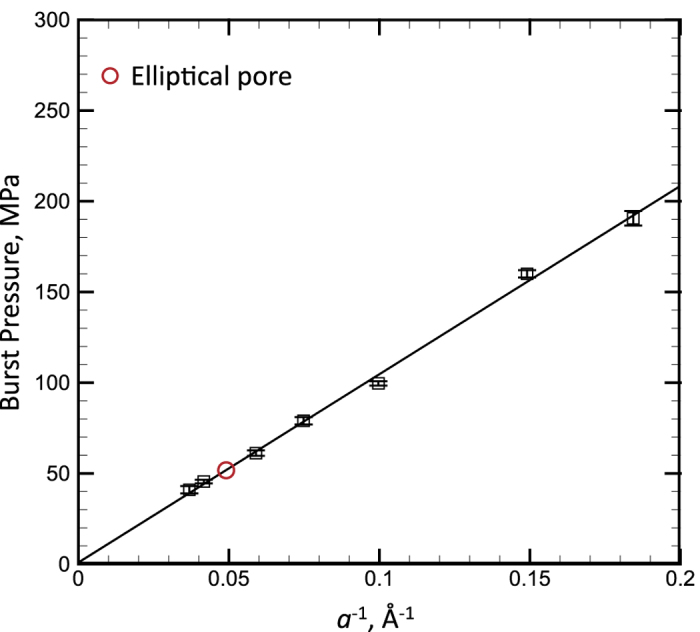
The relationship between the burst pressure and the reciprocal of pore size of water under *T* = 298 K. The solid line is the linear fitting curve.

**Figure 7:**
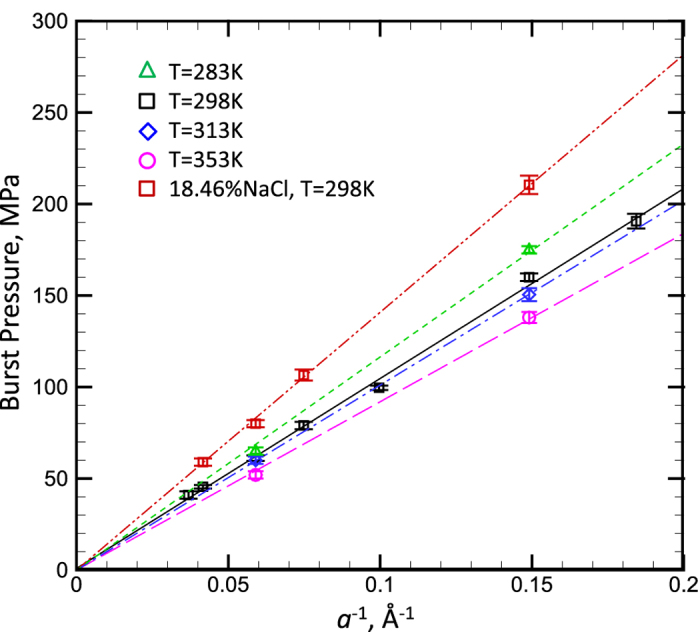
The relationship between the burst pressure and the reciprocal of pore size of water under different temperature as well as adding electrolyte NaCl (the mass concentration is 18.5%). The lines in the figure are the linear fitting curves.
