# Supplementary material for: Effectiveness of the Young-Laplace equation at nanoscale
Source: Sci Rep. 2016 Apr 1;6:23936. doi: 10.1038/srep23936 (PMC4817043; doi:10.1038/srep23936)
Supplement: Supplementary Information [file srep23936-s1.pdf]

Supporting online material for

Effectiveness of the Young-Laplace equation at nanoscale

Hailong Liu and Guoxin Cao*

HEDPS, Center for Applied Physics and Technology, College of Engineering, Peking University, Beijing 100871, China.

**Corresponding author. E-mail address: caogx@pku.edu.cn. Telephone number: 086-01-62756284.*

1. Pressure across water surface

In MD simulations, the piston (the bottom carbon plane) pressure applied on the reservoir (see Figure 2 in the main text) is expressed as the following equation:

$$P_{pis} = \frac{F}{l_x l_y} = \frac{1}{l_x l_y} \sum_{i=1}^{n_P} \sum_{j=1}^{n_w} f_{ij}$$
$$f_{ij} = -\frac{du_{ij}}{dr_{ij}},\ u_{ij} = \varepsilon \left[\left(\frac{\sigma}{r_{ij}} \right)^{12} - 2 \left(\frac{\sigma}{r_{ij}} \right)^{6} \right] \quad \text{(S1)}$$

where l_x, l_y are the lateral sizes of piston, F is the total force applied on the piston, n_p and n_w are the numbers of the atoms of piston and water molecules inside the cut-off distance from piston, f_{ij}, u_{ij} are the interaction force and energy between piston atom i and water molecule j, respectively, which are the functions of the atomic distance r_{ij}, and σ and ε are the L-J potential parameters. Figure S1 shows the relationship between the piston pressure and the density of water reservoir. The solid line in the figure is the equation of state calculated from the bulk water based on the SPC/E model. Thus, the piston pressure matches very well with the water pressure in reservoir.

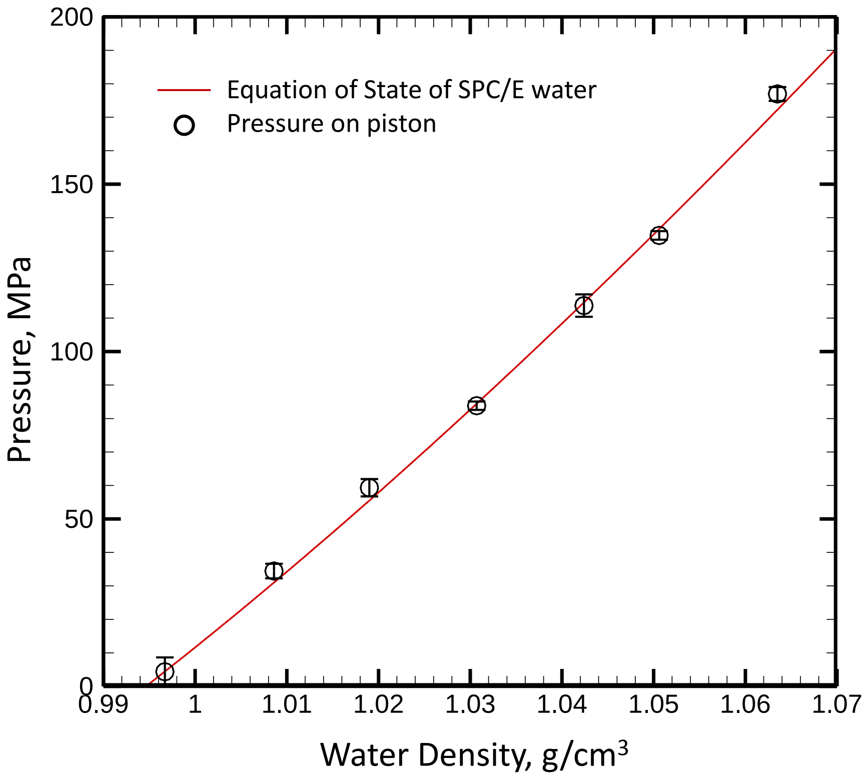

Figure S1. The relationship between the piston pressure and the density of water reservoir. The solid line shows the equation of state of bulk water (SPC/E model) calculated by MD simulations.

After the system is equilibrated, there is a small fluctuation in P_a (e.g., $\Delta P_{pis}/P_{pis} < 10\%$), whereas the mean value of P_{pis} matches very well with the reservoir pressure determined from the equation of state (P_a). In the present work, the pressure in reservoir (P_a) is determined by the equation of state based on the change of average density of reservoir. In the present model, the pressure outside reservoir is zero, and thus, the pressure across the water surface (the water molecules face the pore of top plane) is equal to the water pressure in reservoir ($\Delta P = P_a$). For simplification, the reservoir pressure is defined as ΔP.

2. Curvature of water surface

For the equilibrium water conformation under a given pressure, two principal curvature radii R_1 (measured in *xz* plane) and R_2 (measured in *yz* plane) are defined for water surface, which are calculated from the average distribution of surface water molecules in *xz* and *yz* planes using MD simulations. In these two planes, the water surface boundary can be defined by two circles with the same origin (located in the axis line of nanopore) but different radius: all water molecules on the surface can be enclosed between these two circles. The average radius of these two circles is the curvature radius of water surface, shown as the red solid lines in Figure S2, and the uncertainty of the curvature radius is the half of the difference between the radii of those two circles, which is less than 2%. The curvature radii are the statistic results calculated from the distributions of the *x*, *z* coordinates (or *x*, *y* coordinates) of water molecules based on 3500 snapshots. Figure S2a shows the result of the carbon plane with the hydrophilic L-J parameters (set A, the reported contact angle based which is 65°[1]), in which the angle between the tangential line of the water surface and the carbon plate $\theta < \theta_c$ (θ_c is the contact angle). Figure S2b shows the result of the carbon plate with the hydrophobic L-J parameters (set B, the reported contact angle based which is 110°[1]), in which $\theta = 90°$.

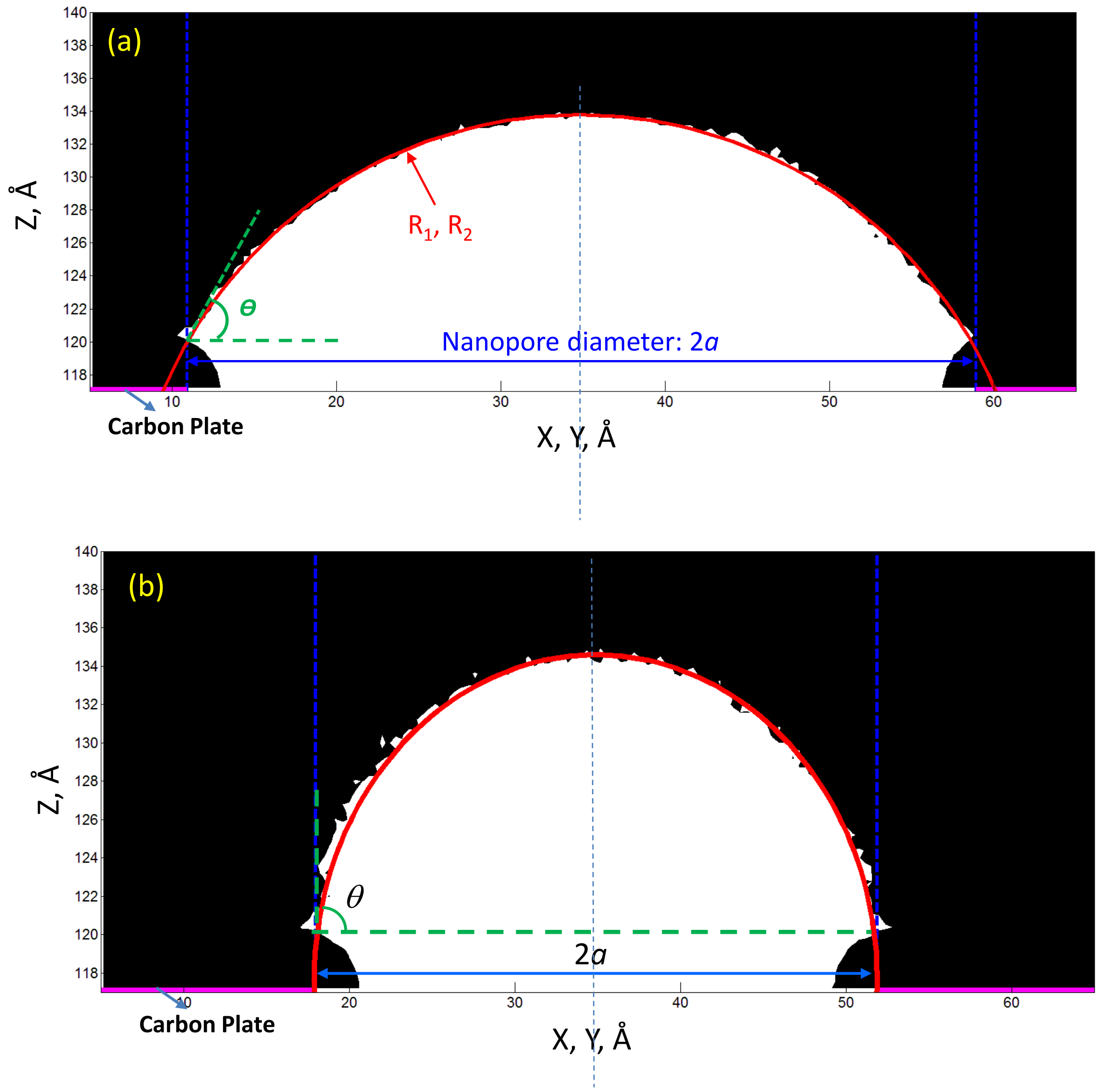

Figure S2. The curvature radius of water surface over nanopore. (a) the carbon plane with the hydrophilic L-J parameters (set A) and $\theta < \theta_c$, ; (b) the carbon plane with the hydrophobic L-J parameters (set B), $\theta = 90°$.

3. The uncertainty analysis of the surface tension

According to Equation (1) (in the main text), the surface tension γ is determined by the applied values of ΔP and the measured values of R_1 and R_2. The distribution of the calculated values of γ with the different values of ΔP and the pore sizes (a) is displayed in Figure S3. The dashed line is the distribution function (determined from 22 data):

$$f(x) = \frac{1}{\sqrt{2\pi\sigma}} \exp\left(-\frac{(x-\mu)^2}{2\sigma^2}\right) \tag{S2}$$

where the mean value μ = 62.6 mN/m and the standard deviation σ = 0.8 mN/m, and thus the

surface tension of water is determined as 62.6 ± 0.8 mN/m in the present work. The uncertainty of the surface tension calculated in the present work is very small, e.g., less than 2%.

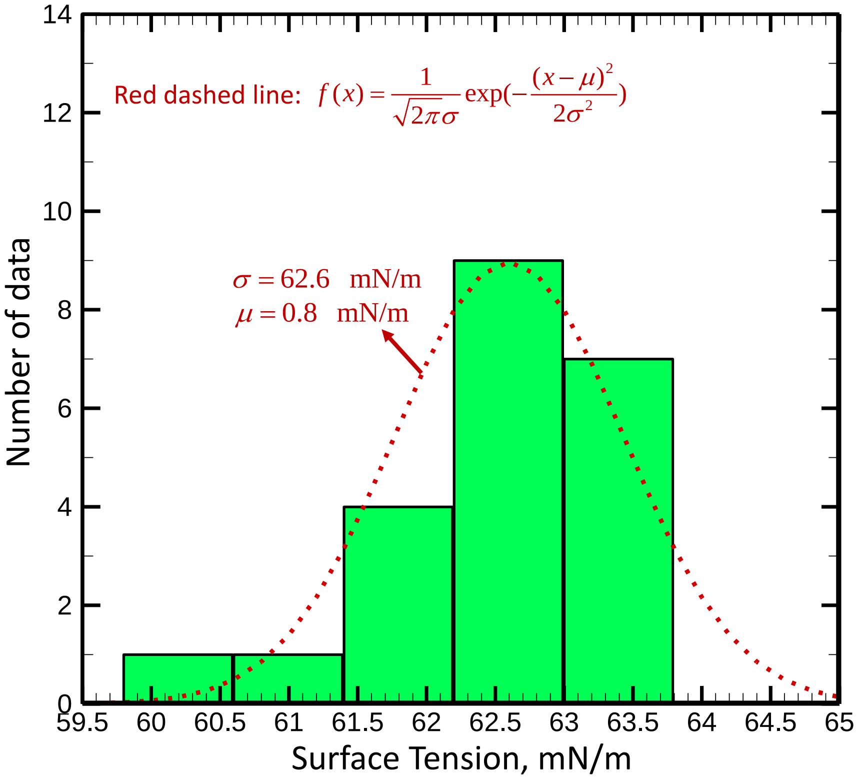

Figure S3. The distribution of the surface tension calculated from the applied and the measured values of R_1 and R_2.

4. The effect of the pore material and the pore shape on surface tension

Figure S4 shows the calculated values of the main curvature radii under different ΔP based on the Set B L-J parameters (ε_{OC} = 0.114333 kcal/mol and σ_{OC} = 0.32751 nm, representing the hydrophobic surface and the contact angle θ_c = 110°[1]) as well as the elliptical pore with the same pore area (Set A L-J parameters). The dashed line in the figure is calculated from Equation 1 in the main text, in which the water surface tension determined in the present work is used (62.6 mN/m). After the pore material is changed from hydrophilic to hydrophobic (the surface contact angle is changed from 65° to 110°), there is no noticeable change in the surface radius of curvature, which means that the surface tension determined is the intrinsic property of water (i.e., insensitive to the solid material property). In addition, when the pore shape is changed from the circular to elliptical, one of the principal radius (R_1) increases but the other decreases (R_2), which leads to the same average principal curvature radius. Figure S5 shows the calculated values of the water surface tension based on the data displayed in Figure S4 using Equation (1) (in the main text).

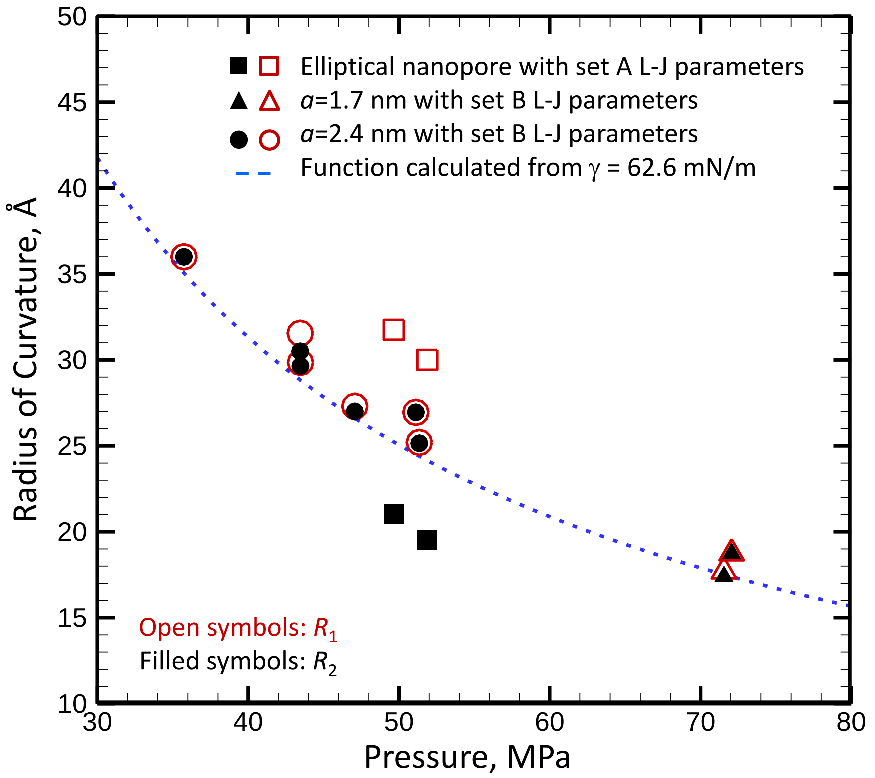

Figure S4. The calculated values of the main curvature radii of water surface based on set B parameters or the elliptical nanopore (set A parameters).

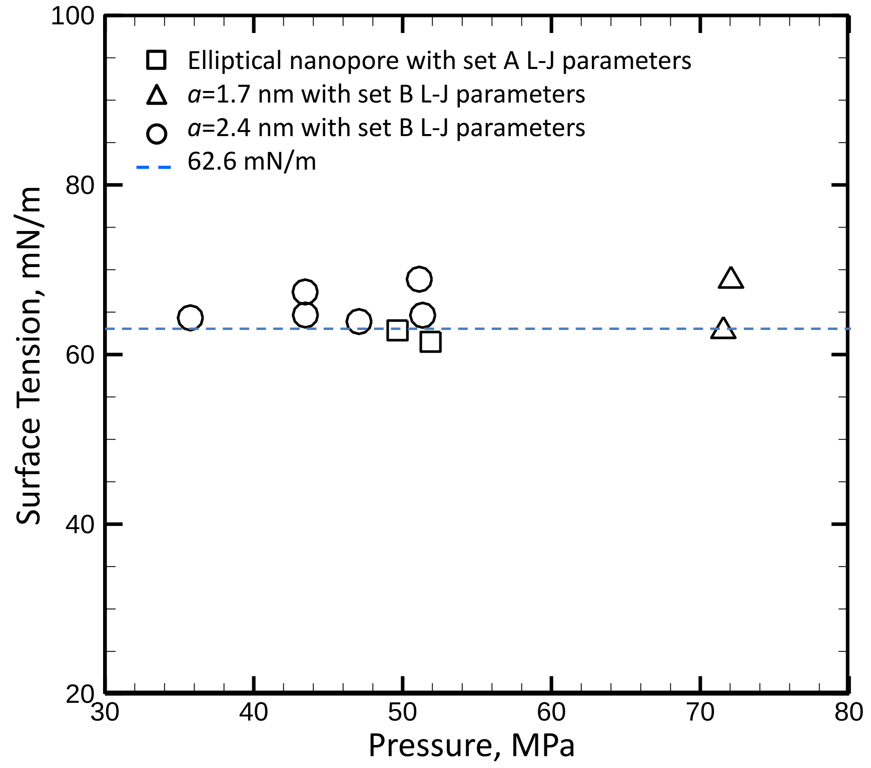

Figure S5. The water surface tension based on the data displayed in Figure S4 using Equation (1) (in the main text).

5. The conformation of water surface

The conformation of water structure can be typically described by the coordination number and the average near O-O distance of water molecules (representing the hydrogen bond structure). Figures S6 and S7 show the average near O-O distance and the coordination number of water surfaces created by 45.2 MPa on the pore (a = 2.4 nm) and 33.6 MPa on the pore (a = 2.7 nm), respectively. Actually, the water surface has a thickness at nanoscale, in which there is a variation in the density, average near O-O distance and coordination number with the radial distance across the surface thickness, as shown in Figures S6 and S7. In the present work, the surface thickness is defined by two dashed lines in the figures: the right line (representing the outer boundary of surface) decided by the water molecule distribution (see Figure S2); the left line shows the location where the average near O-O distance (or the coordination number) begins to converge. The inset figure shows the schematic of water molecule distribution near to the water surface. For the different values of a and ΔP, the average near O-O distances are very similar but there is a small change in the coordination number, and thus, the average near O-O distance of surface is not sensitive to the pore radius and the reservoir pressure. The average near O-O distance (or the coordination number) of the whole water surface is calculated as the mean value of the water molecules between two thin dashed lines, as shown in Figure 5 in the main text.

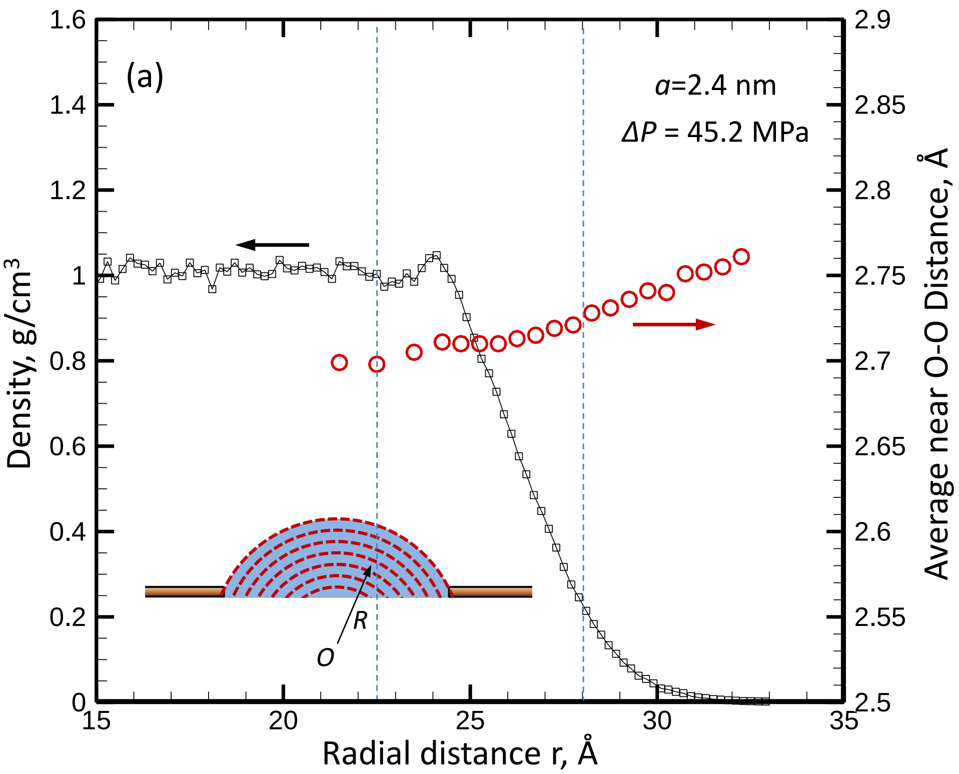

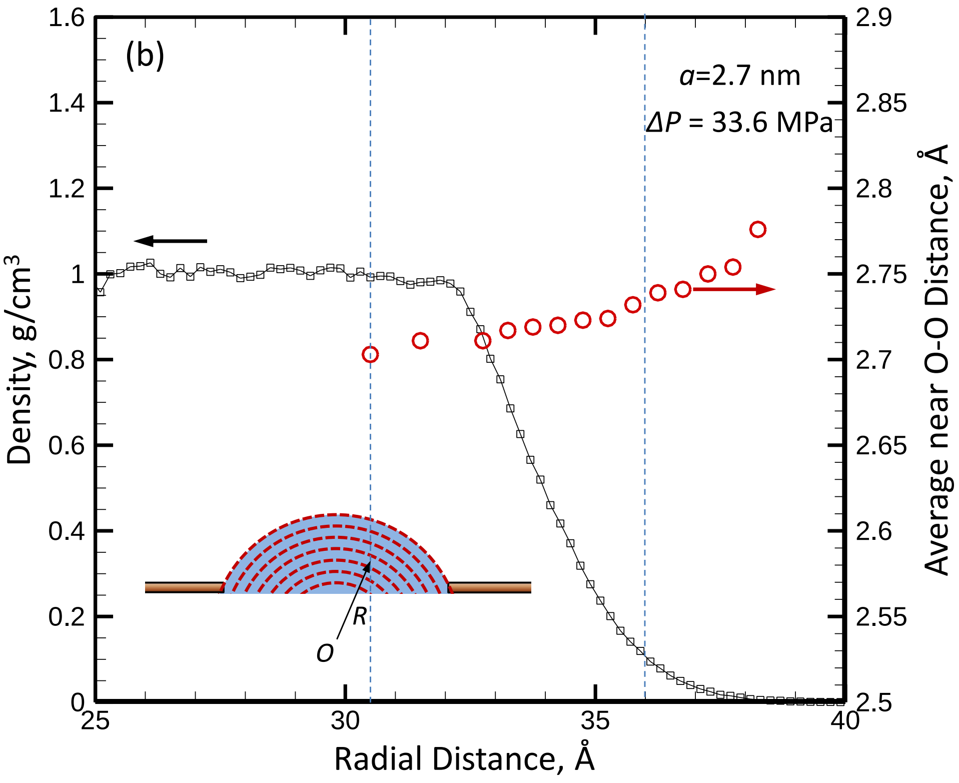

Figure S6. The density and the average near O-O distance of water molecules varying with radial distance from center. (a) *a* = 2.4 nm, *ΔP* = 45.2 MPa; (b) *a* = 2.7 nm, *ΔP* = 33.6 MPa.

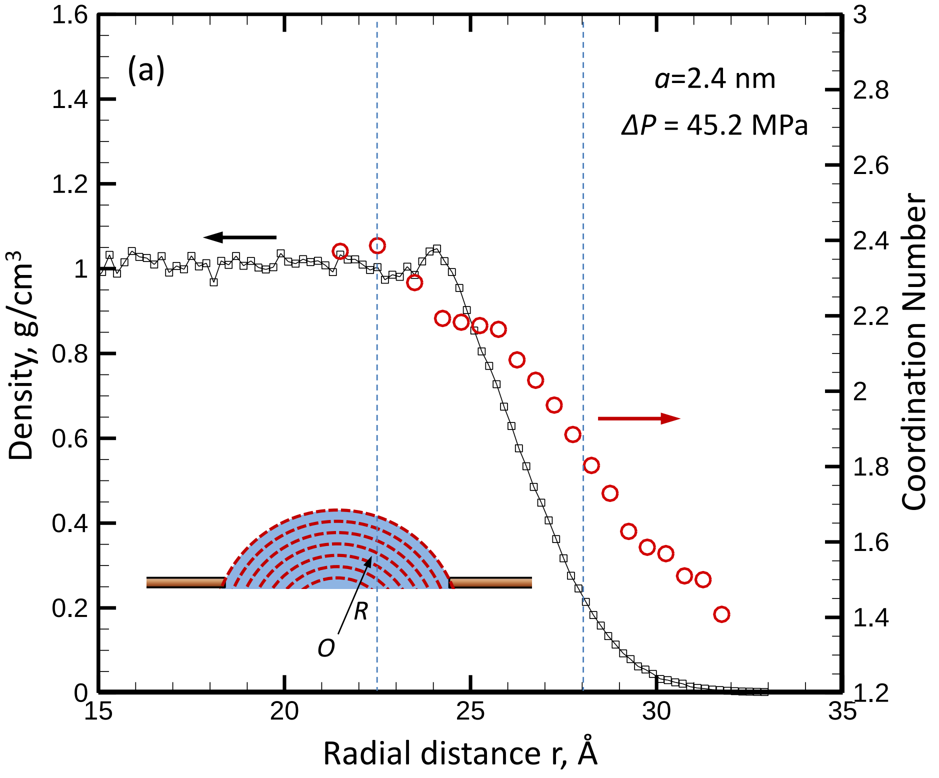

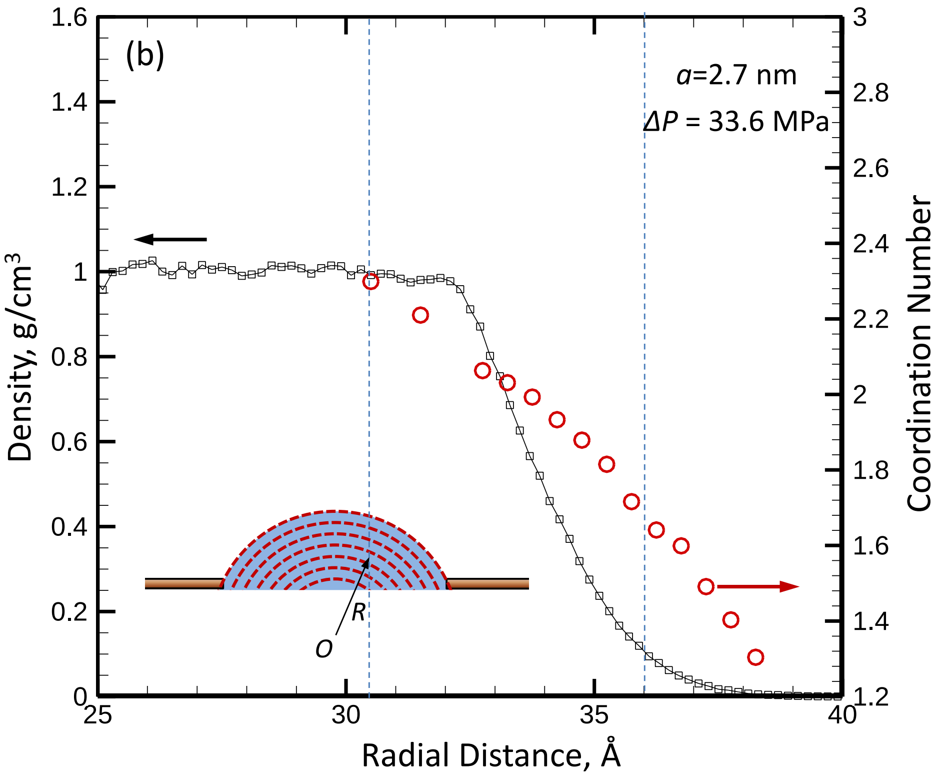

Figure S7. The density and the average coordination of water molecules varying with radial distance from center. (a) a = 2.4 nm, ΔP = 45.2 MPa; (b) a = 2.7 nm, ΔP = 33.6 MPa.

6. The burst pressure of water out of nanopore

According to Equation (1) (in the main text), the curvature radius of water surface decreases with the increase of ΔP, and the curvature radius cannot be less than the pore radius a, at which the water surface is an exact half spherical surface over the pore. There should be no water molecules outside of this half sphere when the water surface is stable, and thus, the number of water molecules outside of this half sphere (N_{out}, also defined as free water molecules) can be used as a criterion for checking the water surface stability: N_{out} will be rapidly increased when the applied pressure is higher than the critical value, which is defined as the burst pressure in the present work. Figure S8 shows the value of N_{out} (a = 1 nm) varying with the MD simulation time under the different applied pressure. From the figure, the burst pressure of water with a = 1 nm is determined as 99.6 ± 0.9 MPa.

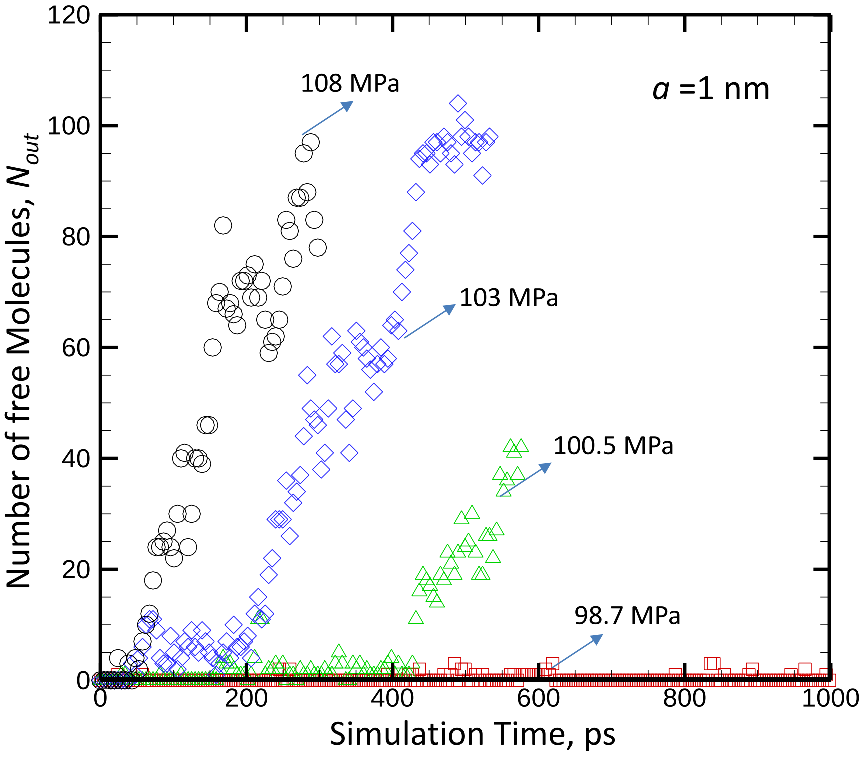

Figure S8. The number of free water molecules outside of the half sphere over the nanopore (N_{out}) under the different pressure varying with the MD simulation time.

7. The infiltration pressure of water into a rigid SWCNT segment

The water reservoir is connected with a rigid SWCNT segment (with the radius a = 1.0 nm), and the water cannot enter into SWCNT initially since the SWCNT is hydrophobic (using set B L-J parameter). In MD simulations, the reservoir pressure P_a is calculated under the different piston displacements, which is calculated based on the same approach described in Section 1 (supporting material). With the increase of the piston displacement, the reservoir pressure P_a increases in the initial stage and then P_a converges to some critical value P_{in}, which is defined as the infiltration pressure of water into SWCNT, as shown in Figure S9.

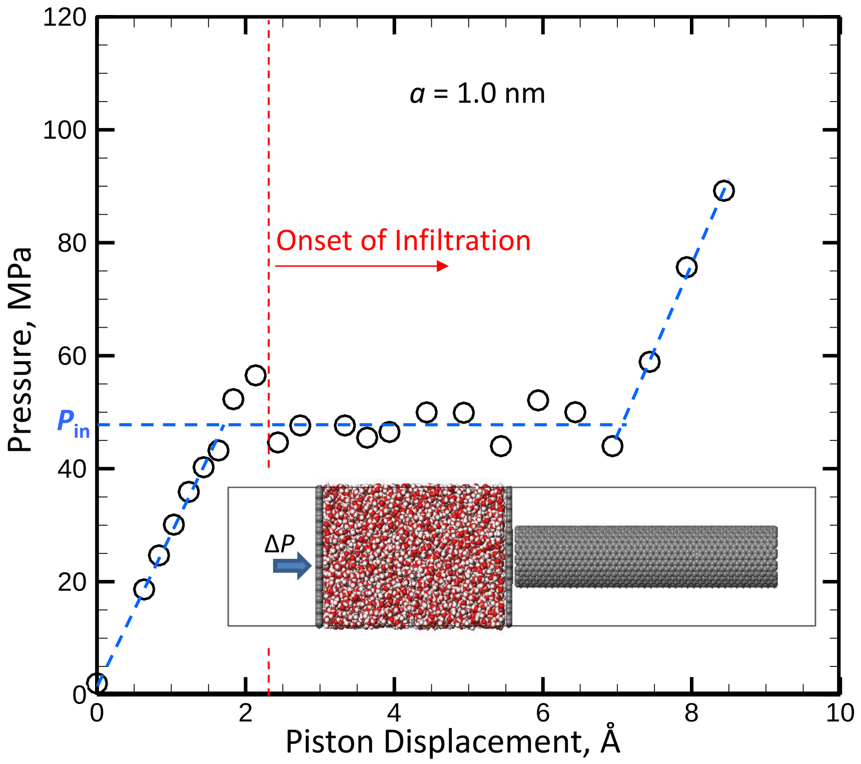

Figure S9. The relationship between the reservoir pressure and the piston displacement. The dashed lines in the figure are the linear fitting curves. The inset figure show the computational model used in MD simulations.

References

1 Werder, T., Walther, J. H., Jaffe, R. L., Halicioglu, T. & Koumoutsakos, P. On the water-carbon interaction for use in molecular dynamics simulations of graphite and carbon nanotubes. *J Phys Chem B* **107**, 1345-1352, (2003).
